# Circulation of 2 Barmah Forest Virus Lineages in Military Training Areas, Australia

**DOI:** 10.3201/eid2612.191747

**Published:** 2020-12

**Authors:** Wenjun Liu, Joanne R. Kizu, David R. Matley, Richard Grant, Fiona J. McCallum, Christopher G. Moller, Tracy L. Carthew, Jun Hang, Ania J. Gubala, John G. Aaskov

**Affiliations:** Australian Defence Force Malaria and Infectious Disease Institute, Enoggera, Queensland, Australia (W. Liu, J.R. Kizu, R. Grant, F.J. McCallum, C.G. Moller, T.L. Carthew);; Enoggera Health Centre, Brisbane, Queensland, Australia (D.R. Matley);; Walter Reed Army Institute of Research, Silver Spring, Maryland, USA (J. Hang);; Australian Defence Science and Technology Group, Fishermans Bend, Victoria, Australia (A.J. Gubala);; Institute of Health and Biomedical Innovation, Brisbane (J.G. Aaskov)

**Keywords:** alphaviruses, arboviruses, Australian military, Barmah Forest virus, genetic sequencing, mosquito-borne diseases, phylogenies, reverse transcription PCR, Togaviridae, viruses, Australia, vector-borne infections

## Abstract

During 2017–2018, Barmah Forest virus was recovered from mosquitoes trapped in military training areas in Australia and from a soldier infected at 1 of these areas. Phylogenies of the nucleotide sequences of the envelope glycoprotein gene E2 and the 3′ untranslated region suggest that 2 lineages are circulating in eastern Australia.

With »15,000 laboratory-confirmed cases over the last decade, Barmah Forest virus (BFV) is the second most common cause of human arboviral disease in Australia, after Ross River virus (RRV) (*1*). BFV is a positive-sense, single-strand, enveloped RNA virus of genus *Alphavirus*, family *Togaviridae*. Other viruses in this genus include chikungunya virus, RRV, Sindbis virus, and Eastern and Western equine encephalitis viruses. BFV was first isolated in 1974 from *Culex annulirostris* mosquitoes trapped near Barmah Forest, northern Victoria, Australia (*2*); the first case of a clinical BFV infection in humans was reported in 1986 (*3*). Since then, BFV has been reported throughout mainland Australia and Papua New Guinea (*4*,*5*). Clinical signs and symptoms of BFV infection, including polyarthritis, arthralgia, and myalgia, are similar to but milder than those of RRV infection (*6**–**7*). Through phylogenetic analyses of the nucleotide sequences of complete E2 envelope protein genes and of the 3′ untranslated region (3′ UTR), we identified 3 BFV lineages. However, we found only 1 example of 2 of the lineages (*5*,*8*). 

RRV caused epidemic polyarthritis outbreaks in military personnel in Australia during and after short military exercises in the Shoalwater Bay Training Area in northeastern Australia in 2016 and 2017 (*9*). The soldier in this study was among personnel who sought treatment during the 2017 outbreak with a suspected RRV infection. Signs and symptoms included rash on the face and body, nausea, headache, fatigue, lethargy, and joint and muscle pain. This retrospective study was approved by the Australian Department of Defence and Department of Veterans’ Affairs Human Research Ethics Committee (DDVA HREC), Joint Health Command Low-Risk Ethical Review Panel (no. 16-021). We obtained formal written consent from the soldier. 

During a retrospective investigation of the outbreak, using PanBio ELISA kits (Abbott, https://www.abbott.com), we detected BFV IgG and IgM, but not RRV IgG and IgM, in convalescent serum samples collected 23, 28, and 38 days after onset of symptoms in the patient. After inoculating 100 µL of the serum into cultures of C6/36 mosquito cells and 2 subsequent passages in this cell line, we did not detect infectious virus in the acute-phase serum sample collected on the day of symptom onset. However, we detected BFV RNA, but not RRV RNA, using a quantitative reverse-transcription PCR assay of RNA extracted from 140 µL of the acute-phase sample using a QiaAMP Viral RNA Mini Kit (QIAGEN, https://www.qiagen.com). BFV E2 RNA was present at 4.2 ×10^6^ copies/mL, with a forward primer 8985F (5′-AGTGTGGCAGTACAACTCCCAAT-3′) corresponding to genome position 8985–9006 and a reverse primer (5′-AAGGCACATGGATCTTTCCTTTC-3′) corresponding to genome position 9036–9058. 

For sequencing, we amplified the E2 and 3′ UTR genes by reverse transcription PCR using primers E2 forward 8205F 5′-GCTGTCTGACCACTACTACCA-3′ and E2 reverse 9833R 5′-GACTTAATCACTACTAAAGATAGCG-3′, and 3′ UTR forward 10923F 5′-TCCATCCATCTCTACTACCG-3′ and reverse poly-T 5′-TTTTTTTTTTTTTTTTTTTTG-3′ designed from the nucleotide sequence of the prototype BFV strain (BH2193) and synthesized by Sigma (https://www.sigmaaldrich.com). The PCR amplifications were performed using *pfu* DNA polymerase (Promega, https://www.promega.com), which has 3′–5′ exonuclease proofreading activity. The PCR amplicons were separated by agarose gel electrophoresis and recovered using a rapid gel extraction system (QIAGEN). Sequencing was performed at the Australian Genome Research Facility as described elsewhere (*10*). Sequences were assembled and edited using Geneious version 11.2 (https://www.geneious.com). The human isolate was named SWBTA40/2017. Two strains of BFV (TullyA/2017 and WBTA/2018) isolated from mosquitoes collected in the Tully and Wide Bay military training areas in Queensland, eastern Australia, in 2017 and 2018 (Table 1), as well as 7 BFV strains collected previously in Australia during 1974–2015, were also sequenced in the same manner. All sequences were submitted to GenBank (accession no. MK169381-6 and MH618666). Seven BFV E2 gene sequences within GenBank, including that of a recent isolate from Papua New Guinea (*5*), were included in phylogenetic analyses. 

**Table 1:** Details of BFV strains used for phylogenetic study of virus lineages circulating in military training areas, Australia*

No.	Isolate	Location	Isolation year	Hosts	GenBank accession no.
1	BFVBH2193	Barmah Forest, Victoria	1974	*Culex annulirostris*	NC_001786
2	BFVC583	Beatrice Hill, Northern Territory	1978	*Culicoides marksi*†	MK169381
3	BFV16313	Charleville, Queensland	1974	*Cx. annulirostris*	MK169382
4	BFVC530SAB8	Beatrice Hill, Northern Territory	1975	*Cx. annulirostris.*	MK169383
5	BFV19493	Charleville, Queensland	1976	*Aedes normanensis*	MK169384
6	BFV19418BF	Charleville, Queensland	1976	*Cx. annulirostris*	MK169385
7	BFV16287	Charleville, Queensland	1974	*Ae. normanensis*	MK169386
8	BFVMIDI4	Brisbane, Queensland	2015	*Homo sapiens*	MH618665
9	BFV-TullyA	ADF Tully Beach training area, Queensland	2017	*Verrallina spp*	MK169387
10	BFVWBTA	ADF Wide Bay training area, Queensland	2018	*Ae. vigilax*	MH618666
11	BFVSWBTA40	Shoal Water Bay training area, Queensland	2017	*Homo sapiens*	MK169388
12	BFV18295	Australia	1993	*Cx. annulirostris*	JX855115
13	BFV145357	Australia	2008	*Cx. annulirostris*	JX855116
14	BFV106287	Australia	1995	*Ae. vigilax*	JX855117
15	BFV80504	Australia	2006	*Ae. vigilax*	JX855118
16	BFV76707	Australia	2006	*Ae. procax*	JX855119
17	BFV78362	Australia	NR	NR	JX855120

*ADF, Australian Defence Force; BFV, Barmah Forest virus; NR, Not recorded. †Biting midge species.

The SWBTA40/2017 E2 comprised 1263 nt corresponding to nucleotides 8290–9552 of the genomic RNA of the BFV prototype strain BH2193. The sequence similarity among all 17 E2 sequences we examined was remarkably high, with an overall divergence of <4.1%. The nucleotide sequence of SWBTA40/2017 was most closely related to that of the BFV prototype strain BH2193, but differed from it at 4 sites, 8692 (A®G), 8835 (U®C), 9108 (A®G), and 9427 (U®C), resulting in nucleotide divergence of 0.32% (Table 2). The A®G substitution at 8692 resulted in an amino acid substitution of Asn®Asp and the U®C substitution at 9427 resulted in a Ser®Pro amino acid change. SWBTA40/2017 E2 gene diverged from TullyA/2017 in 24 (1.9%) nt positions and from WBTA/2018 in 25 (1.98%) nt positions (Table 2). The A®G substitution at 9197 resulted in a Ser®Asn change and a U®C substitution at 9433 resulted in a Phe-to-Leu change in both the TullyA and WBTA BFV strains. 

**Table 2:** Nucleotide and deduced amino acid differences in E2 of the Barmah Forest virus from the Shoalwater Bay Training Area compared with the prototype strain and strains isolated by the Australian Defence Force, Australia*

Characteristic	Strain (GenBank accession no.)			
	BH2193 (NC_001786)	SWBTA40 (MK169388)	WBTA (MH618665)	TullyA (MK169387)
Geographic origin	Northern Victoria	Central Queensland	Central Queensland	Central Queensland
Year of isolation	1974	2017	2018	2017
Nucleotide no.†	Changes in nt seq (aa)	Changes in nt seq (aa)	Changes in nt seq (aa)	Changes in nt seq (aa)
8313	C	NC	U	U
8394	C	NC	U	U
8412	C	NC	U	U
8466	U	NC	C	C
8472	U	NC	C	C
8556	A	NC	U	U
8619	U	NC	C	C
8628	A	NC	G	G
8631	A	NC	G	G
8673	C	NC	U	U
8692	A (Asn)	G (Asp)	NC	NC
8745	U	NC	C	C
8835	U	C	NC	NC
8865	G	NC	A	A
9108	A	G	NC	NC
9197	G (Ser)	NC	A (Asn)	A (Asn)
9229	A (Ser)	NC	G (Gly)	NC
9246	U	NC	C	C
9295	C	NC	U	U
9315	A	NC	U	U
9342	U	NC	A	A
9354	A	NC	U	U
9427	U (Ser)	C (Pro)	NC	NC
9433	U (Phe)	NC	C (Leu)	C (Leu)
9510	C	NC	U	U

*A, adenine; aa, amino acids; C, cytosine; G, guanine; NC, no changes; nt seq, nucleotide sequence; U, uracil †Nucleotide numbers correspond to those of the prototype BH2193 strain.

The SWBTA40/2017 3′ UTR comprised 443 nt corresponding to nucleotides 11047–11488 of the prototype strain BH2193 but with a single-nucleotide insertion at position 11128 (Figure 1); it differed from that of the TullyA/2017 strain by 110 nt and the WBTA/2018 strain by 114 nt (Figure 1), with multiple insertions/deletions (indels) occurring in the recent TullyA/2017 and WBTA/2018 strains. The noticeable differences were the finding of 2 large indels at nucleotide positions 3–27 and 205–263 of the 3′ UTR (Figure 1). The potential influence of these indels on BFV replication and transmission warrants further study because the 3′ UTR region plays critical roles in alphaviral gene expression, replication, protein translation, and host tropism (14). 

**Figure 1:**
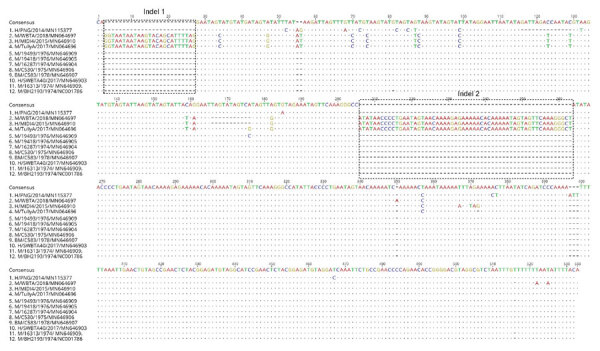
Nucleotide alignment of 3′ untranslated region sequences of Barmah Forest virus strains from Australia with that of the prototype BH2193 strain using muscle alignment method in Geneious version 11.2 (https://www.geneious.com). The dots indicate the consensus sequence of Barmah Forest virus strains, whereas letters in individual sequences indicate nucleotide substitutions. Dashes indicate insertions/deletions. The naming convention of the strains is name of host/strain/year of isolation/GenBank accession number. H, humans; M, mosquitoes; BM, biting midges.

We derived phylogenies using Geneious version 11.2 with neighbor joining, maximum-likelihood, and Bayesian analysis for 17 complete E2 and twelve 3′ UTR sequences. E2 sequences segregated into 3 lineages with strong bootstrap support (Figure 2, panel A), instead of the 1 (*8*) or 2 (*5*) lineages reported previously. The lineages are numbered chronologically; the strain from the central province of Papua New Guinea from 2014, which appears to be the oldest, was denoted as lineage I (*5*). The isolates described in this study (SWBTA40/2017, TullyA/2017, and WBTA/2018) were placed into 2 distinct lineages, lineages II and III. The conclusion about 3 lineages was supported by the analysis of the 3′ UTR regions (Figure 2, panel B). 

**Figure 2:**
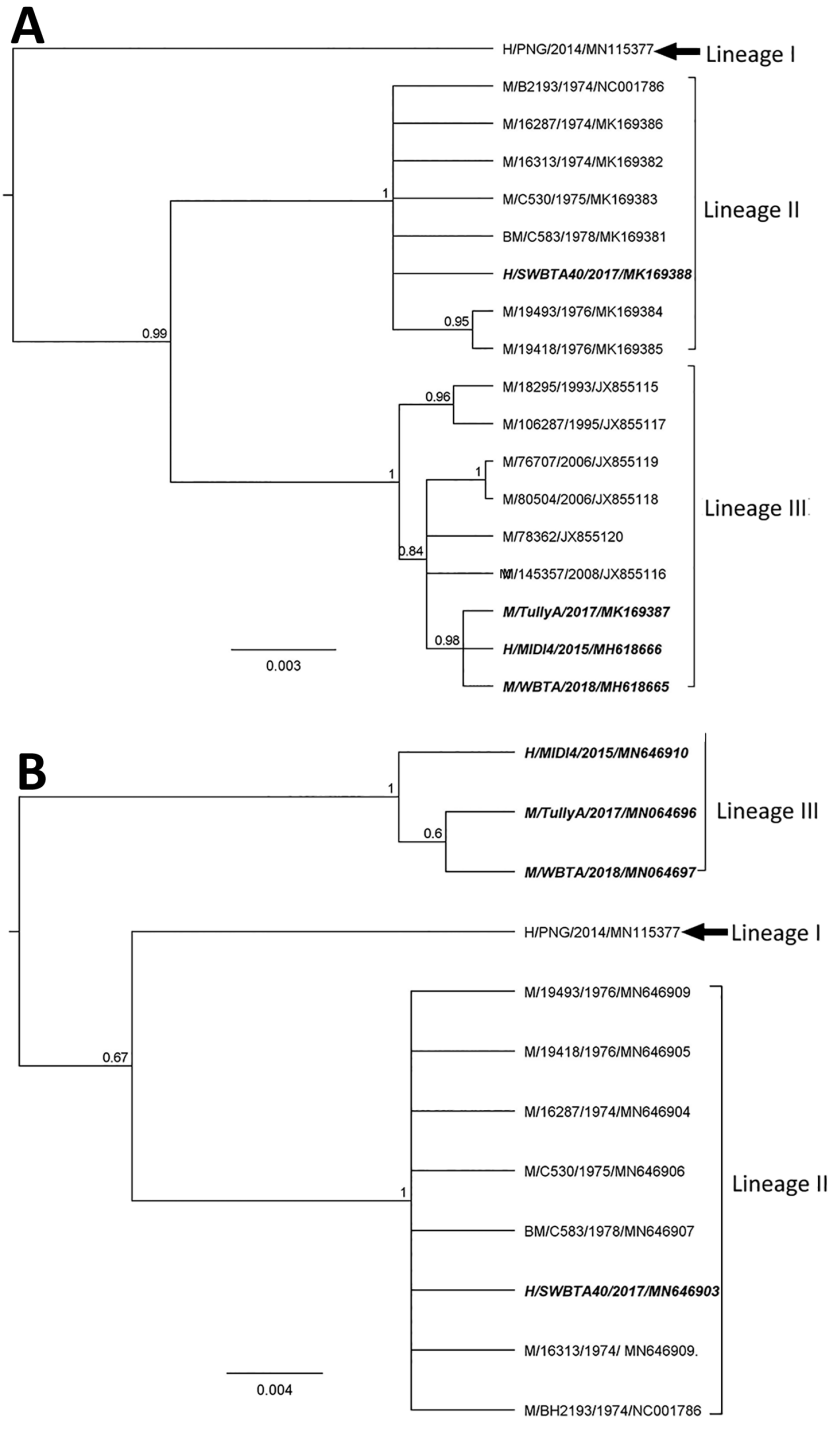
Phylogenies based on 17 complete BFV E2 sequences (1,263 bp) (A) and twelve 3′ untranslated region sequences (B) classify Barmah Forest virus isolates into 3 distinct lineages. We used Bayesian phylogenetic analysis method in Geneious version 11.2 (https://www.geneious.com) to analyze the aligned E2 and 3′ untranslated region sequences, applying the Hasegawa-Kishino-Yano plus gamma substitution model with a gamma molecular clock model of uniform branch lengths, a chain length of 1 million, and a 10% burn-in length. The naming convention of the strains is name of host/strain/year of isolation/GenBank accession number. Scale bar indicates the length of the branches of each tree. H, humans; M, mosquitoes; BM, biting midges.

All amino acid substitutions were located in the C domain of the E2 protein in areas that are involved in interaction with other proteins (E1, capsid, and 6k), as well as in the process of budding of alphavirus envelope proteins from host cell membranes (*11*,*12*). BM/C583/1978 in lineage II was isolated from *Culicoides* midges rather than mosquitoes and may reflect BFV in a blood meal rather than this insect being a vector for BFV. Nonetheless, given the position of BFV in the phylogeny of alphaviruses, exploration of vectors other than mosquitos for this virus might be warranted.

Given the relatively recent detection of BFV in western Australia (*13*,*14*) and the basal position in phylogenetic trees of the only isolate of BFV from Papua New Guinea, the cocirculation of 2 lineages of BFV in eastern Australia points to a poor understanding of population dynamics and evolution in this virus. The ongoing replacement of strains of RRV and the appearance of strains with epidemic potential (*15**)* suggests that BFV may warrant more detailed virological surveillance. 
